# 2298. Clinical and Demographic Characteristics of COVID-19 in Pediatric Patients in the United States

**DOI:** 10.1093/ofid/ofac492.136

**Published:** 2022-12-15

**Authors:** Milan Ho, Zachary M Most, Marlon Diaz, Julia A Casazza, Bhaskar Thakur, Sameh Saleh, Madison Pickering, Trish M Perl, Christoph U Lehmann, Richard J Medford, Robert W Turer

**Affiliations:** University of Texas Southwestern Medical Center, Dallas, TX; University of Texas at Southwestern Medical Center, Dallas, Texas; University of Texas Southwestern Medical Center, Dallas, TX; University of Texas Southwestern Medical Center, Dallas, TX; University of Texas Southwestern Medical Center, Dallas, TX; Children's Hospital of Pennsylvania, Philadelphia, Pennsylvania; University of Chicago, Chicago, Illinois; University of Texas Southwestern Medical Center, Dallas, TX; University of Texas Southwestern Medical Center, Dallas, TX; University of Texas Southwestern Medical Center, Dallas, TX; University of Texas Southwestern Medical Center, Dallas, TX

## Abstract

**Background:**

The percentage of children infected with COVID-19 has outpaced that of adults. As children >5 years are now eligible to receive vaccines, it is necessary to understand the effect of vaccination in the context of demographic characteristics, clinical factors, and variants on pediatric COVID-19 illness severity.

**Methods:**

We conducted a descriptive study of patients ≤18 years from the Optum® COVID-19 electronic health record dataset. Patients were included if positive for COVID-19 by polymerase chain reaction or antigen testing for the first time from 3/12/2020 to 1/20/2022. We compare race and ethnicity, age, gender, US region of residence, vaccination status, body mass index (BMI), pediatric comorbidity index (PCI) (Sun, Am. J. Epidemiol. 2021), and predominant variant (by time and region) with 2-tailed t-test, multi-category chi-square test, and odds ratios (R version 4.1.2; α = 0.05). PCI is a validated comorbidity index predicting hospitalization in pediatric patients.

**Results:**

Of all pediatric patients in our dataset, 165,468 (13.2%) tested positive for COVID-19. 3,087 (1.9%) were hospitalized, 1,417 (0.9%) were admitted to the ICU, 1545 (0.9%) received respiratory support, and 31 (0.02%) died, comparable to AAP-reported hospitalization and mortality rates in US children. Patients with severe outcomes were more likely to be younger, non-Caucasian, from the US South, unvaccinated, and have a higher PCI (Figure 1). Excluding non-severe outcomes, rates of death and ICU admission were higher in 0–4-year-olds compared to 5–11 or 12–18-year-olds (Figure 2). All patients receiving at least one dose of the vaccine survived. The odds ratio of a severe outcome is 0.11 (95% CI 0.07–0.16) in fully vaccinated patients compared to unvaccinated patients. The odds ratio of a severe outcome is 0.55 (95% CI 0.49–0.63) in partially vaccinated patients compared to unvaccinated patients.

Demographic and clinical characteristics of pediatric patients with COVID-19

Relative proportion of clinically severe outcomes within age groups, excluding non-severe outcomes

**Conclusion:**

In this large population, incidence rate of severe outcomes from COVID-19 in pediatric patients was higher among non-Caucasian patients, living in the South, with underlying comorbid illness, and those not yet eligible for vaccination. These findings reinforce the need for a vaccine for younger patients and targeted vaccine outreach to racial and ethnic minorities and children with chronic conditions.

**Disclosures:**

**Christoph U. Lehmann, MD**, Celanese: Stocks/Bonds|Markel: Stocks/Bonds|Springer: Honoraria|UTSW: Employee.

